# Effects of psychological care in patients with endometriosis: A systematic review protocol

**DOI:** 10.1097/MD.0000000000014772

**Published:** 2019-03-08

**Authors:** Juan Gao, Han-Qiao Liu, Yan Wang, Ya-Li Shang, Fang Hu

**Affiliations:** a Gynecology Department, Second Affiliated Hospital of Shaanxi University of Chinese Medicine, Xianyang; b Gynecology Department, College of Pharmacy, Hubei Medical College, Shiyan, China.

**Keywords:** effect, endometriosis, psychological care, randomized controlled trial

## Abstract

**Background:**

This systematic review will be proposed for investigating the effects of psychological care (PC) in patients with endometriosis.

**Methods:**

We will search the following 7 electronic databases from inception to the present: MEDLINE, EMBASE, SinoMed, Web of Science, Cochrane Library, Chinese Biomedical Literature Database, and China National Knowledge Infrastructure. We will include randomized controlled trials for evaluating the effects of PC in patients with endometriosis. Cochrane risk of bias tool will be used to evaluate the methodological quality for each included study. Two authors will independently carry out the study selection, data extraction, and methodological quality evaluation. Any disagreements will be solved by a third author through discussion.

**Results:**

This proposed systematic review will use high-quality evidence-based medicine to evaluate the efficacy and safety of PC for endometriosis. The primary outcomes include depression and anxiety. The secondary outcomes consist of pain intensity, health-related quality of life, and adverse events.

**Conclusion:**

The findings of this study will provide convincing evidence to determine whether PC therapy is an effective and safe intervention for patients with endometriosis.

**PROSPERO registration number:**

PROSPERO CRD42019123292.

## 1 Introduction

Endometriosis is a very common gynecological disorder,^[1–3]^ which often impacts reproductive-aged women, and is one of the most common reasons for infertility.^[4–6]^ Previous study has reported that its prevalence ranges from 5% to 10% in premenopausal women, and it can affect about 35% of those with subfertility.^[7]^ It is characterized as the presence of ectopic endometrial tissue outside the uterus.^[8–10]^ This disorder often manifests as dyspareunia, cyclic menstrual pain, chronic pelvic pain, and dyschezia.^[11–13]^ Patients who experience such condition often complain that it severely affects their quality of life, as well as the psychological conditions, such as depression and anxiety.^[14–16]^

A variety of clinical studies have reported that psychological care (PC) can be effectively used to treat psychological conditions in patients with endometriosis.^[17–24]^ However, up to the present, no systematic review and meta-analysis have been conducted to explore the effects of PC for patients with endometriosis. Therefore, in the present proposed systematic review, we will specifically assess the effects of PC for the patients with endometriosis.

## 2 Methods

### 2.1 Review registration

This systematic review protocol has been registered in PROSPERO with number of CRD42019123292. It has been reported abiding to the Preferred Reporting Items for Systematic Reviews and Meta-analyses (PRISMA) Protocols statement guidelines.^[25]^

### 2.2 Eligibility criteria for included studies

#### 2.2.1 Types of studies

This study will include randomized controlled trials (RCTs) that have assessed all forms of PC for patients with endometriosis. However, the other studies, such as non-clinical trials, non-control studies, non-RCTs, and quasi-RCTs will not be considered for inclusion.

#### 2.2.2 Types of participants

All patients with endometriosis are clinically diagnosed as having psychological conditions, such as depression and anxiety will be fully considered for inclusion without any restrictions.

#### 2.2.3 Type of interventions

The participants in the experimental group can receive any forms of PC interventions. However, the combination of PC with other treatments will not be considered. The participants in the control group can receive any therapies, except the PC.

#### 2.2.4 Type of outcomes

The primary outcome includes depression and anxiety, as measured by depression anxiety stress scales or any other instruments. The secondary outcomes consist of pain intensity, as assessed by visual analog scale or any other scales; health-related quality of life, as evaluated by 36-item short form survey, or any other tools; as well as any adverse events.

### 2.3 Strategy of literature searches

The following electronic databases will be searched from their inceptions to the present without language restrictions: MEDLINE, EMBASE, SinoMed, Web of Science, Cochrane Library, Chinese Biomedical Literature Database, and China National Knowledge Infrastructure. Any potential studies for assessing the effects of PC for endometriosis will be fully considered for inclusion. The sample of detailed search strategy for Cochrane Library is presented in Table 1. Identical search strategies will also be applied to other electronic databases.

**Table 1:**
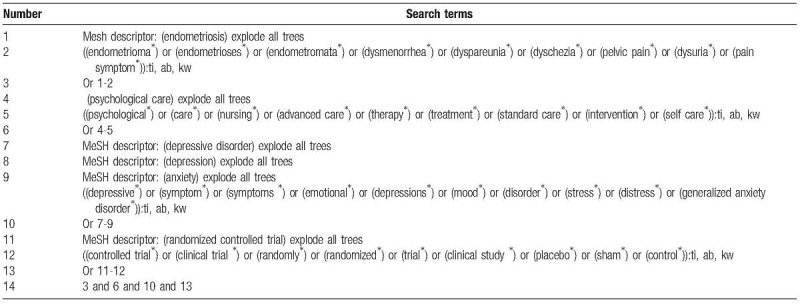
Search strategy applied in the Cochrane Library database.

Additionally, clinical registry, and reference lists of included RCTs and relevant reviews will also be searched. Besides, we will also search grey literature, such as dissertations to avoid missing any potentially eligible studies.

### 2.4 Data collection

#### 2.4.1 Study selection

Two authors will independently scan the title and abstract for each study initially. Then, full-texts will be read to further identify the potentially eligible studies for inclusion. All study selection will be carried out according to the predefined eligibility criteria, and the whole process will be presented in the PRISMA flowchart. If any divergences regarding the study selection exist between 2 authors, a third author will be consulted through discussion.

#### 2.4.2 Data extraction

Two authors will independently extract the data from each included according to the predefined data collection form. Any divergences between 2 authors will be resolved through discussion with a third author. The form comprises of title, first author, publication year, location, patient characteristics, diagnosed criteria, eligibility criteria, number of patients, details of randomization, blinding, interventions in both experimental and control groups, all outcome measurements, and adverse events.

#### 2.4.3 Dealing with missing data

Any missing data or insufficient information will be contacted by the primary authors of original studies by email. If those data are not available, then we will just analyze the available data. Moreover, we will also discuss its impacts as a limitation.

### 2.5 Risk of bias assessment for included studies

Two authors will independently assess the methodological quality for each included RCT by using the Cochrane Collaboration Tool. Any disagreements regarding the risk of bias evaluation between 2 authors will be consulted a third author and will be solved through discussion. This tool comprises of 7 items, and each item will be judged as high risk of bias, unclear risk of bias, or low risk of bias.

### 2.6 Treatment effect measurement

The results of continuous data will be measured by using the mean difference or standardized mean difference with 95% confidence intervals (CIs), while the results of dichotomous data will be measured by using risk ratio with 95% CIs.

### 2.7 Heterogeneity assessment

Heterogeneity among eligible studies will be assessed by using *I*^2^ test. Acceptable heterogeneity will be considered if *I*^2^ ≤50%. Otherwise, significant heterogeneity will be regarded if *I*^2^ >50%.

### 2.8 Data synthesis

RevMan 5.3 software will be used for all data pooling and meta-analysis conducting. If acceptable heterogeneity is detected, a fixed-effect model will be used, and meta-analysis will be conducted if it is possible. If substantial heterogeneity is identified, a random-effect model will be used, and meta-analysis will be carried out according to the results of subgroup analysis. If there is reasonable heterogeneity after the subgroup analysis, then meta-analysis will be performed. Otherwise, data will not be pooled, and meta-analysis will not be conducted. Instead, we will just report a narrative summary.

### 2.9 Subgroup analysis

Subgroup analysis will be carried out to detect any potential reasons that cause the significant heterogeneity. It will be conducted according to the different characteristics of study or patients, treatments, controls, and outcome measurements.

### 2.10 Sensitivity analysis

Sensitivity analysis will be performed to identify the robustness of the results by taking away low-quality studies.

### 2.11 Publication bias

In this systematic review, funnel plot^[26]^ and Egg regression^[27]^ will be conducted if sufficient eligible studies (more than 10 RCTs) are included.

## 3 Discussion

Endometriosis is a common cause of infertility in females. A variety of managements are used to treat this condition, such as medication, physical therapy, and PC. Although lots of clinical studies have shown that PC can effectively reduce the psychological symptoms, such as depression, and anxiety, its efficacy has not assessed scientifically or systematically.^[17–24]^ To our best knowledge, no systematic review or meta-analysis has addressed to assess the effects and safety of PC for patients with endometriosis that have been published. It is crucial to determine whether PC is an effective intervention for relieving psychological condition in patients with endometriosis. The present systematic review and meta-analysis will provide important information to benefits patients, clinicians, as well as the health policy-maker with a comprehensive understanding of the effects of PC intervention.

## Author contributions

**Conceptualization:** Juan Gao, Yan Wang, Fang Hu.

**Data curation:** Juan Gao, Ya-Li Shang, Fang Hu.

**Formal analysis:** Juan Gao, Han-Qiao Liu, Yan Wang, Ya-Li Shang.

**Funding acquisition:** Fang Hu.

**Investigation:** Fang Hu.

**Methodology:** Juan Gao, Han-Qiao Liu, Yan Wang, Ya-Li Shang.

**Project administration:** Fang Hu.

**Resources:** Juan Gao, Han-Qiao Liu, Yan Wang, Ya-Li Shang, Fang Hu.

**Software:** Juan Gao, Han-Qiao Liu.

**Supervision:** Yan Wang, Ya-Li Shang.

**Validation:** Han-Qiao Liu, Fang Hu.

**Visualization:** Juan Gao, Ya-Li Shang.

**Writing – original draft:** Juan Gao, Yan Wang, Ya-Li Shang, Fang Hu.

**Writing – review and editing:** Juan Gao, Han-Qiao Liu, Yan Wang, Ya-Li Shang, Fang Hu.
